# Sonographic Association of Umbilical Cord Thickness in the Third Trimester of Pregnancy With Fetal Outcome in a Rural Indian Population

**DOI:** 10.7759/cureus.100310

**Published:** 2025-12-29

**Authors:** Sayali Alaspurkar, Shila Shelke, Poonam Shivkumar, Shuchi Jain, Ankita V Golhar

**Affiliations:** 1 Obstetrics and Gynaecology, Mahatma Gandhi Institute of Medical Sciences, Wardha, IND

**Keywords:** adverse neonatal outcome, diameter of the cord, obstetric ultrasonography, third trimester screening, umbilical cord

## Abstract

Objectives

To assess the association between third-trimester umbilical cord thickness measured on ultrasonography and key neonatal outcomes (birth weight, NICU admission, APGAR score, and meconium-stained liquor) and to examine the association between umbilical cord thickness and selected maternal characteristics, including body mass index.

Design

This is a prospective observational study conducted over 24 months.

Material and methods

A total of 126 antenatal women with singleton pregnancies between 32 and 40 weeks of gestation with normal amniotic fluid index (8-24 cm) and three vessel unit umbilical cord attending a rural tertiary care hospital were enrolled. Exclusion criteria included medical comorbidities affecting fetal outcomes such as gestational diabetes, hypertensive disorders, anemia, maternal chronic illnesses, and fetal congenital anomalies; fetal growth restriction, prematurity, and prelabour rupture of membranes. Umbilical cord thickness was measured sonographically at a free-floating loop approximately one cm from placental insertion using a five MHz curvilinear probe. Measurements were taken thrice and averaged for accuracy. The cord parameters were classified into lean (<10th percentile), normal (10th-90th percentile), and thick (>90th percentile) groups based on study percentiles. Fetal outcomes measured at delivery included birth weight (with low birth weight defined as <2.5 kg), APGAR scores at five minutes (with less than 7 considered low), presence of meconium-stained liquor, and NICU admissions. Statistical correlations were assessed using chi-square tests and Pearson/Spearman correlation coefficients, with p-values <0.05 considered significant.

Results

Among participants, 19(15%) had lean cord thickness, 89 (70.7%) had a normal cord, and 18 (14.3%) had a thick cord. Lean cords showed a significant association with adverse outcomes: 14 (73.6%) neonates with lean cords had low birth weight compared with 10 (11.2%) in the normal group. NICU admission rates were higher in the lean group (11, 57.8%) than in the normal (6, 6.8%) and thick cord (2, 11.2%) groups. Meconium-stained liquor was present in seven (36.9%) of lean cords versus five (5.7%) in normal cords. Low APGAR scores (<7 at five minutes) were significantly more frequent in lean cords. Maternal body mass index was positively correlated with umbilical cord thickness (p=0.0006).

## Introduction

Low birth weight (LBW) remains a significant global health concern, contributing substantially to perinatal morbidity and mortality. It is estimated that 15-20% of all births worldwide, accounting for more than 20 million newborns annually, are LBW, which is strongly linked to adverse neonatal outcomes. In India, neonatal mortality rates range from 18 per 1,000 births in urban areas to 27.5 per 1,000 in rural regions, highlighting the urgent need for effective fetal surveillance [1].

The well-being and growth of the fetus depend on various factors, including genetic influences, maternal characteristics, and placental and umbilical cord structure and function [2]. The umbilical cord serves as a vital lifeline, transporting oxygenated, nutrient-rich blood to the fetus via the umbilical vein and returning deoxygenated blood through its two arteries. The umbilical cord’s morphology, particularly its thickness, reflects the quantity of Wharton’s jelly and vessel integrity - key components influencing nutrient delivery and fetal growth [3,4].

Ultrasonography enables non-invasive, antenatal evaluation of umbilical cord morphology from as early as 8-10 weeks of gestation [5,6]. The thickness of the umbilical cord tends to increase as pregnancy advances, reaching a peak around 32 weeks, followed by a plateau phase. These sonographic parameters potentially provide critical information about fetal growth patterns and intrauterine well-being [4].

Previous studies have linked lean umbilical cords (defined as thickness below the 10th percentile) with fetal growth restriction (FGR), LBW, and adverse perinatal outcomes such as increased neonatal intensive care unit (NICU) admissions and low Apgar scores at birth [7,8]. Conversely, thick cords (above the 90th percentile) have been associated with macrosomia [9]. Despite their clinical relevance, routine antenatal assessment of umbilical cord thickness remains underutilised, especially in the Indian context.

This study aims to address this gap by investigating the sonographic correlation of umbilical cord thickness in the third trimester of pregnancy with fetal outcomes, including birth weight, APGAR score at five minutes, presence of meconium-stained liquor, and NICU admission. Early identification of fetuses at risk through these parameters could enhance antenatal surveillance, guide timely clinical interventions, and ultimately improve neonatal health outcomes.

## Materials and methods

Study design and setting

This prospective observational study was conducted over a 24-month period (June 2022 to July 2024) in the Department of Obstetrics and Gynecology at Mahatma Gandhi Institute of Medical Sciences, Sewagram, India. A total of 126 antenatal women with singleton pregnancies between 32 and 40 weeks of gestation attending the outpatient clinic for delivery were enrolled after obtaining written informed consent.

Sample size

The calculated sample size was 126, based on the literature and taking into account local context with expected test sensitivity of 50%, specificity of 95%, and prevalence of low birth weight of 10%, with 30% precision, 95% confidence interval, and 15% drop out rate.

Inclusion criteria

Pregnant women with a singleton live fetus in the third trimester (32-40 weeks of gestation) with a normal amniotic fluid index (8-24) and the presence of a normal three-vessel umbilical cord with reliable gestational age established by last menstrual period or early ultrasound.

Exclusion criteria

Exclusion criteria were the following: maternal medical disorders, including gestational diabetes mellitus (GDM), hypertensive disorders, anaemia, and other chronic diseases (renal, cardiac, pulmonary), fetal congenital anomalies, clinical evidence of fetal growth restriction (FGR) or macrosomia prior to study enrollment, prelabour rupture of membranes, and cases complicated by spontaneous preterm labour after enrolment.

Methodology

After informed consent, sociodemographic and obstetric history were recorded. Ultrasound examination was performed using a Philips Healthcare machine HD15 equipped with a 5 MHz curvilinear probe. All ultrasound examinations were performed by trained sonographers who were blinded to pregnancy outcomes at the time of umbilical cord thickness assessment.

**Figure 1:**
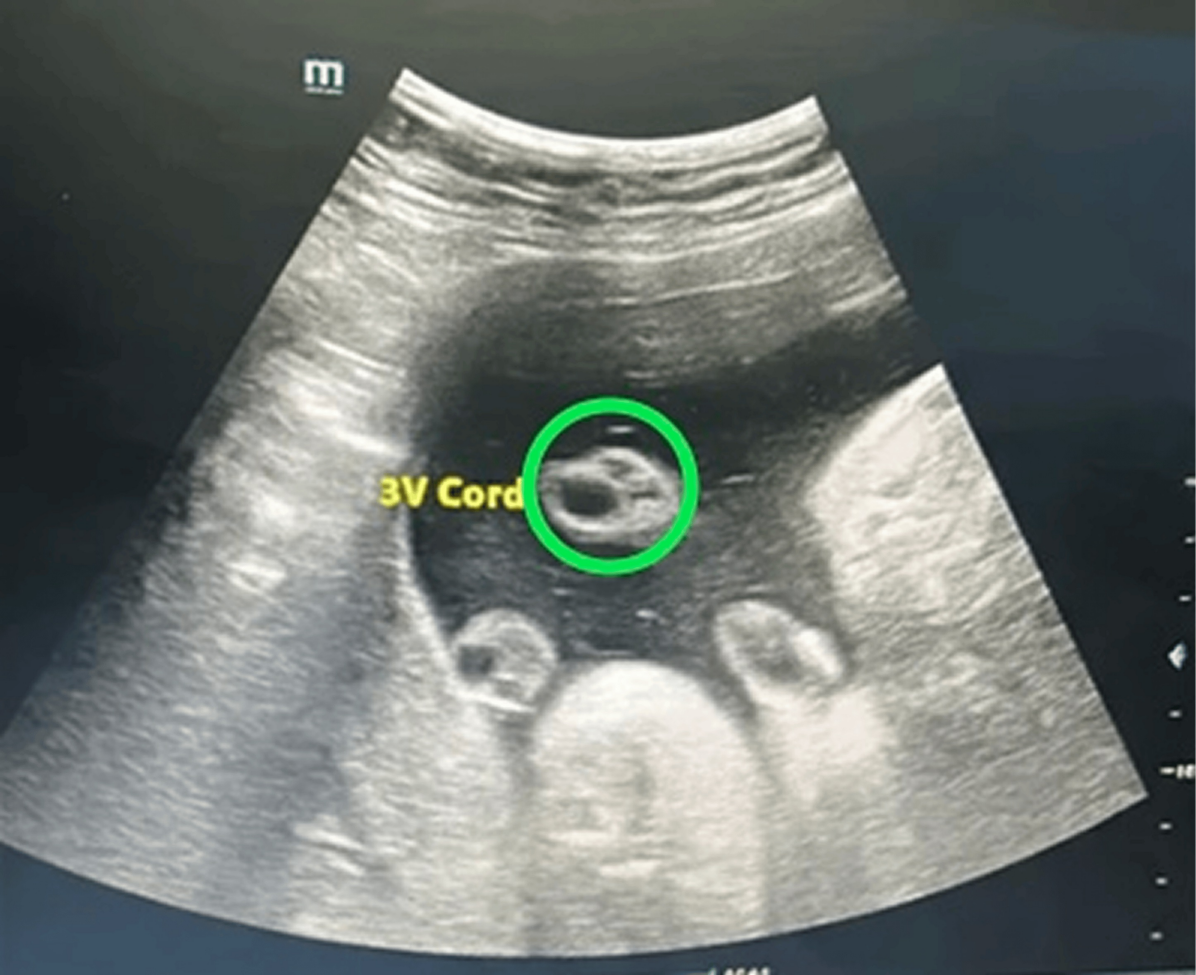
Three-vessel umbilical cord

Umbilical cord thickness was measured sonographically in a free-floating loop of the cord approximately 1 cm from its placental insertion (refer to Figure 1). Color Doppler was employed to confirm cord position and vessel identification. Each parameter was measured three times for accuracy, and measurements were taken from outer-to-outer diameter. The mean value was used for analysis. Umbilical cord thickness was measured in a transverse (cross-sectional) plane. All measurements were performed according to the International Society of Ultrasound in Obstetrics and Gynecology (ISUOG)/myometrial uterus sonographic assessment (MUSA)-recommended sonographic principles.

**Figure 2:**
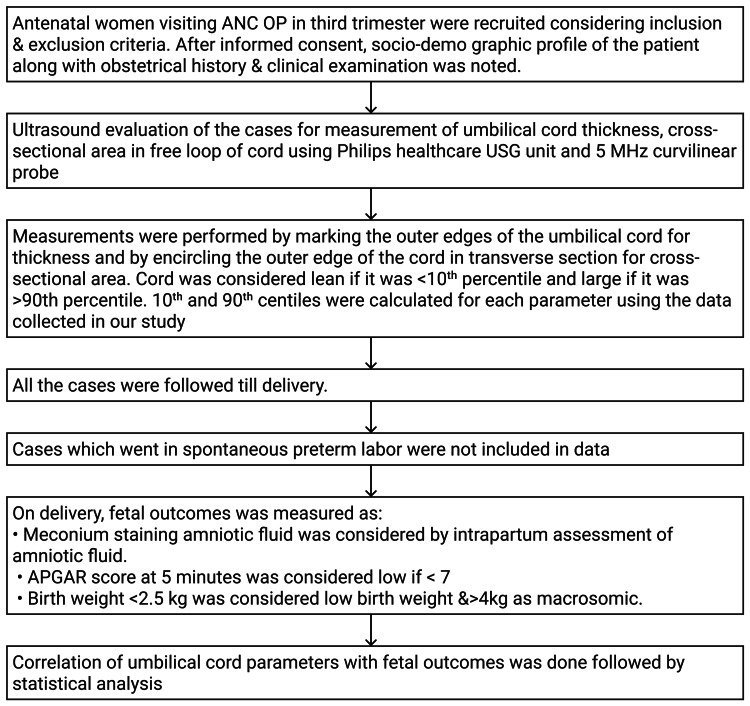
Study design

Umbilical cord thickness was defined as the maximum diameter across the outer cord edges. Percentile cut-offs (<10th percentile defined as lean cords, 10th-90th percentile as normal, >90th percentile as thick cords) were calculated based on study population data. The study design is shown in Figure 2.

Fetal outcomes assessed

The parameters on which the fetal outcomes were judged were the following: birth weight (low birth weight defined as <2.5 kg and macrosomia as more than 4 kg), APGAR score at five minutes (<7 considered low), presence of meconium-stained liquor during labor (clinically assessed), NICU admission (yes/no), and indication for admission (birth length of the baby).

Statistical analysis

Data were entered into Microsoft Excel (Microsoft® Corp., Redmond, WA) and analyzed using Statistical Product and Service Solutions (SPSS, version 21.0; IBM SPSS Statistics for Windows, Armonk, NY). Continuous variables were summarized as means and standard deviations; categorical variables as frequencies and percentages.

Chi-square tests were used to assess associations between categorical variables, such as cord thickness categories and neonatal outcomes. Pearson’s correlation coefficient or Spearman’s rank correlation was used to assess relationships between continuous variables. For comparison among more than two groups, one-way ANOVA or Kruskal-Wallis tests were used for continuous variables, while chi-square or Fisher’s exact tests were applied for categorical variables, as appropriate.

A p-value of less than 0.05 was considered statistically significant.

## Results

Table 1 depicts the maternal and pregnancy characteristics. The study involved 126 pregnant women aged between 21 and 30 years, with nearly 85 (67.4%) having a normal BMI. The participants were a mix of primigravida (55, 43.6%) and multigravida, with varied educational backgrounds mostly up to higher secondary or graduation level. This demographic profile reflects a typical maternity population suitable for assessing umbilical cord parameters.

**Table 1:** Baseline demographic and obstetric characteristics of the study participants (N=126) Booked patients were those who had received regular antenatal care at the study institute (≥3 antenatal visits/at least one in each trimester)with all routine investigations done, whereas registered patients were those who were registered at the institute but had irregular or limited antenatal follow-up.

Parameter	Category	Number	Percentage (%)
Age (years)	≤ 20	4	3.2
	21–25	51	40.4
	26–30	61	48.5
	31–35	8	6.3
	36–40	2	1.6
Body Mass Index (BMI, kg/m²)	Underweight (≤18.5)	20	15.9
	Normal (18.6–22.9)	85	67.4
	Overweight (23–24.9)	17	13.5
	Obese (25–30)	4	3.2
Educational Status	< 10th grade	12	9.5
	10th grade	33	26.3
	12th grade	40	31.7
	Graduate	40	31.7
	Postgraduate	1	0.8
Parity	Primigravida	55	43.6
	Gravida 2	41	32.5
	Gravida 3	23	18.2
	Gravida > 3	7	5.5
Booking Status	Booked	57	45.2
	Registered	64	50.8
	Not registered	5	4

**Figure 3:**
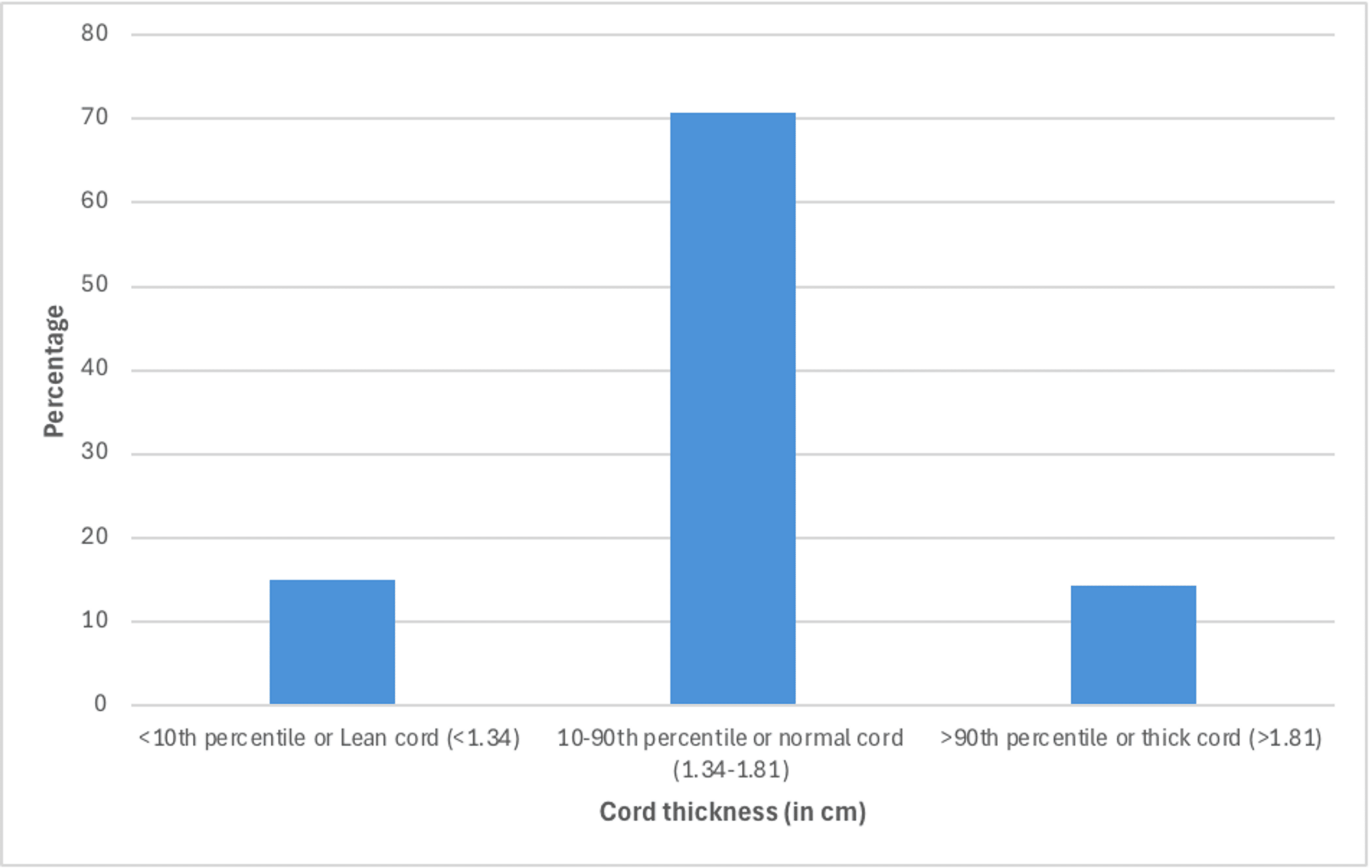
Distribution of cases according to umbilical cord thickness on USG (N=126) USG: ultrasonography

Table 2 and Figure 3 show the distribution of cases according to umbilical cord thickness on ultrasonography. It shows that the majority of cases (89, 70.7%) had cord thickness between the 10th and 90thpercentile, 19 (15.0%) cases had cord thickness of less than the 10th percentile, and 18 (14.3%) cases had cord thickness of more than the 90thpercentile.

**Table 2:** Distribution of cases according to umbilical cord thickness on ultrasonography (in cm) USG: ultrasonography

UC thickness on USG (in cm)	Number	Percentage
<10th percentile or Lean cord (<1.34)	19	15.0
10-90th percentile or normal cord (1.34-1.81)	89	70.7
>90th percentile or thick cord (>1.81)	18	14.3
Total	126	100.0

Table 3 shows all the fetal parameters that were assessed to see the correlation of umbilical cord thickness with different parameters.

**Table 3:** Combined birth outcomes (N=126) LSCS: lower segment caesarean section; NVD: normal vaginal delivery

Parameter	Category	Number	Percentage (%)
Gender	Male	64	50.7
	Female	62	49.3
Birth Weight (g)	≤ 2500 (Low Birth Weight, LBW)	37	29.3
	> 2500-4000	89	70.7
APGAR Score at 5 minutes	< 7 (Low)	2	1.4
	≥ 7	124	98.6
Meconium Staining	Present	17	13.5
	Absent	109	86.5
NICU Admission	Yes	19	15.1
	No	107	84.9
Mode of Delivery	LSCS	68	54.0
	NVD	58	46.0

Table 4 shows that there was a statistically significant association of umbilical cord thickness with BMI. The BMI was directly related to the umbilical cord thickness, while there was no statistically significant association of maternal age and gravidity. 

**Table 4:** Association between umbilical cord thickness and maternal age, gravidity, and BMI

Cord Thickness (cm)	Frequency N(%)	Maternal age (Mean ± SD)	BMI Mean (kg/m^2^)	Gravidity (Mean ± SD)
Lean cord (<1.34)	19 (15%)	25.6 ± 3.9	18.41 ± 2.53	1.7 ± 0.61
Normal cord (1.34-1.81)	89 (70%)	23.19 ± 5.32	20.53 ± 2.65	1.82 ± 0.73
Thick cord (>1.81)	18 (14.3%)	24.33 ± 3.82	23.62 ± 3.52	1.75 ± 0.57
p value		0.0792	0.000629	0.649
Significance		NS	S	NS

Table 5 shows that 14 (73.6%) of those with UC thickness <10th percentile had low birth weight (LBW) as compared to five (26.4%). There was a statistically significant association of the umbilical cord thickness with LBW.

**Table 5:** Association between umbilical cord thickness and birth weight (N=126)

Birth weight (in gm)	UC Thickness (<10th percentile)	UC Thickness (10-90th percentile)	UC Thickness (>90th percentile)	p value
≤2500	14 (73.6%)	10 (11.2%)	13 (72.2%)	<0.0000001
>2500-4000	5 (26.4%)	79 (88.8%)	5 (27.8%)	
Total	19 (100.0%)	89 (100.0%)	18 (100.0%)	

Table 6 shows that 11 (57.8%) of those with UC thickness <10th percentile were admitted to the NICU as compared to eight (42.9%). There was a statistically significant association of the umbilical cord thickness with NICU admissions, suggesting a direct association of lesser umbilical cord thickness with NICU admissions.

**Table 6:** Association between umbilical cord thickness with NICU admissions (N=126) NICU: neonatal intensive care unit

NICU Admission	UC Thickness (<10th percentile)	UC Thickness (10-90th percentile)	UC Thickness (>90th percentile)	p value
Yes	11 (57.8%)	6 (6.8%)	2 (11.2%)	<0.0000001
No	8 (42.2%)	83 (93.2%)	16 (88.8%)	
Total	19 (100.0%)	89 (100.0%)	18 (100.0%)	

**Figure 4:**
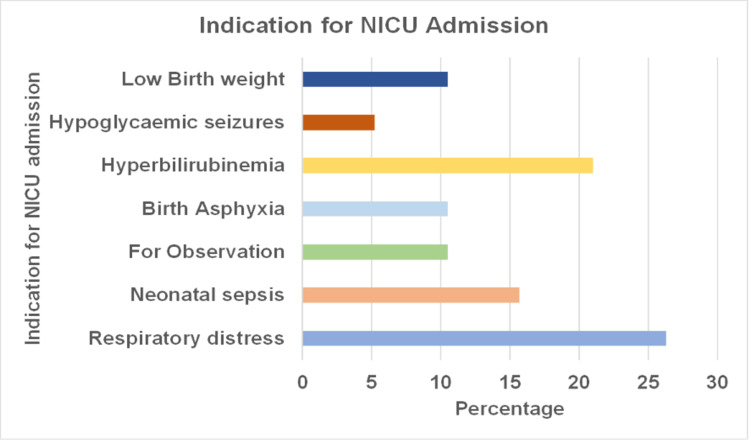
Distribution of cases according to indications for NICU admission (N=19)

Figure 4 shows that most of the cases (5, 26.3%) admitted to the NICU were admitted for respiratory distress, four (21.0%) had hyperbilirubinemia, three (15.7%) had neonatal sepsis, two (10.5%) cases each were admitted for birth asphyxia, LBW, and for observation, respectively. Meanwhile, one case was admitted for hypoglycaemic seizures.

Table 7 shows that there was a statistically significant difference in the UC thickness and meconium staining, indicating that there were more incidences of meconium-stained liquor with abnormal umbilical cords.

**Table 7:** Association between umbilical cord thickness and meconium staining

Meconium Staining	UC Thickness (<10th percentile)	UC Thickness (10-90th percentile)	UC Thickness (>90th percentile)	p value
Present	7 (36.9%)	5 (5.7%)	5 (27.8%)	0.0002305
Absent	12 (63.1%)	84 (94.3%)	13 (72.2%)	
Total	19 (100.0%)	89 (100.0%)	18 (100.0%)	

Table 8 shows that the APGAR score at five minutes was significantly lower in those with cord thickness <10th percentile.

**Table 8:** Association between umbilical cord thickness and APGAR score (N=126)

APGAR Score at 5 mins	UC Thickness (<10th percentile)	UC Thickness (10-90th percentile)	UC Thickness (>90th percentile)	p value
7	1	1	0	<0.0000001
8	0	104	20	

Table 9 describes the correlations of umbilical cord thickness with key fetal outcomes. There was a significant association between lean umbilical cord thickness and LBW and NICU admissions. For example, 14 (73.6%) neonates with lean cord thickness were LBW versus only 10 (11.2%) in the normal group. NICU admissions were 11 (57.8%) in lean cord thickness cases versus only six (6.8%) for normal cords. Meconium-stained liquor was over six times more common in lean cords compared to normal. Low APGAR scores, though rare overall, were predominantly observed in the lean cord group. Thick cords did not show consistent adverse correlations. These findings emphasize lean cord measurements as strong predictors of fetal growth restriction and neonatal morbidity.

**Table 9:** Association between umbilical cord thickness with key outcomes

Outcome	Umbilical Cord Thickness < 10th Percentile (%)	10th–90th Percentile (%)	> 90th Percentile (%)	p-value
Low Birth Weight (≤ 2500 g)	73.6 (14/19)	11.2 (10/89)	72.2 (13/18)	< 0.000001
NICU Admission	57.8 (11/19)	6.8 (6/89)	11.2 (2/18)	< 0.000001
Meconium Staining	36.9 (7/19)	5.7 (5/89)	27.8 (5/18)	0.00023
Low APGAR (<7 at 5 min)	5.26 (1/19)	1.12 (1/89)	0	< 0.001

Limitations

The study was conducted in a single tertiary care center with a moderate sample size, which may limit generalizability. Only third-trimester measurements were taken, and fetal Doppler or placental pathology correlation was beyond the scope. Longer-term neonatal follow-up data were not available. Due to the absence of validated third-trimester Indian reference nomograms, internally derived percentiles were used. Confounders, such as smoking status, and detailed nutritional variables were not determined.

## Discussion

This prospective observational study evaluated the sonographic correlation of umbilical cord thickness with fetal outcomes in 126 antenatal women in the third trimester. The findings demonstrate a strong association between lean umbilical cords - defined as thickness below the 10th percentile - and adverse neonatal outcomes, including LBW, increased NICU admissions, low APGAR scores, and meconium-stained amniotic fluid. These results emphasize the clinical relevance of umbilical cord morphology as a non-invasive predictor of fetal well-being.

The distribution of umbilical cord parameters in this study aligns with expected physiological changes, whereby cord thickness increases with gestational age until around 32 weeks and then plateaus, consistent with prior reports by Raio et al. and Barbieri et al [5,10]. This plateau likely reflects stable fetal growth and Wharton’s jelly composition in late gestation. Lean cords likely indicate reduced Wharton’s jelly or compromised vascular integrity, which can restrict nutrient and oxygen delivery, thereby predisposing to FGR and LBW [11]. Our results show that 73.6% of neonates with lean cords had LBW, strongly supporting this pathophysiological link [12].

A significant positive correlation between maternal BMI and umbilical cord thickness found in this study underscores the impact of maternal nutritional and physiological status on placental and fetal development. This corroborates findings by Richardson et al. showing larger placental size and higher birth weights in mothers with higher BMI [13]. However, maternal age and parity showed no significant association with cord parameters, focusing attention on modifiable maternal factors such as nutrition.

Notably, our data show lean cord parameters are also linked with markers of perinatal stress and morbidity. NICU admissions were significantly higher (57.8%) in lean cord cases, as were incidences of meconium-stained liquor and low APGAR scores. These findings confirm previous studies indicating lean cords as markers of compromised fetal oxygenation and distress [12,14]. Thick cords (>90th percentile), while occasionally associated with macrosomia, did not consistently predict adverse outcomes in our cohort, reflecting the more complex and variable implications of increased cord size [15].

The lack of significant association between cord parameters and birth length suggests that umbilical cord morphology primarily reflects fetal mass and well-being rather than linear growth, aligning with the understanding that length is less sensitive to placental compromise than weight [8,16].

The novelty of this study lies in its focus on the Indian antenatal population, where routine assessment of umbilical cord thickness is not standard practice [17]. This research contributes important normative data to the predictive value of these sonographic parameters for fetal outcomes in this demographic context [8,10]. Considering the high burden of LBW and neonatal morbidity in India, incorporating routine umbilical cord assessment into third-trimester ultrasonography protocols could substantially improve early identification of fetuses at risk.

## Conclusions

The study concluded that thin umbilical cord diameter in the third trimester, as assessed by ultrasonography, is significantly associated with placental insufficiency, leading to LBW and adverse neonatal outcomes. There is a strong positive association between umbilical cord thickness and birth weight and NICU admissions, indicating that a smaller cord size is associated with fetal growth restriction and increased neonatal complications. Maternal BMI showed a direct correlation with umbilical cord thickness, whereas maternal age and parity did not significantly affect cord parameters. Since umbilical cord parameters stabilize after 32 weeks, measuring umbilical cord thickness during routine antenatal scans after this period provides a valuable, easy, and cost-effective tool for screening and predicting fetal growth and perinatal outcomes. This can enable early detection of fetal growth restriction, facilitate close fetal surveillance, timely interventions, and appropriate referral to higher care centres, ultimately improving fetal well-being and perinatal health.

## References

[1] Wang HS, Hung SC, Peng ST (2004). Mesenchymal stem cells in the Wharton's jelly of the human umbilical cord. Stem Cells.

[2] Ferguson VL, Dodson RB (2009). Bioengineering aspects of the umbilical cord. Eur J Obstet Gynecol Reprod Biol.

[3] Qureshi F, Jacques SM (1994). Marked segmental thinning of the umbilical cord vessels. Arch Pathol Lab Med.

[4] Sun Y, Arbuckle S, Hocking G, Billson V (1995). Umbilical cord stricture and intrauterine fetal death. Pediatr Pathol Lab Med.

[5] Raio L, Ghezzi F, Di Naro E, Gomez R, Franchi M, Mazor M, Brühwiler H (1999). Sonographic measurement of the umbilical cord and fetal anthropometric parameters. Eur J Obstet Gynecol Reprod Biol.

[6] Ghezzi F, Raio L, Di Naro E, Franchi M, Balestreri D, D'Addario V (2001). Nomogram of Wharton's jelly as depicted in the sonographic cross section of the umbilical cord. Ultrasound Obstet Gynecol.

[7] Dudiak CM, Salomon CG, Posniak HV, Olson MC, Flisak ME (1995). Sonography of the umbilical cord. Radiographics.

[8] Narayan R, Saaid R, Pedersen L, Hyett J (2015). Ultrasound assessment of umbilical cord morphology in the first trimester: a feasibility study. Fetal Diagn Ther.

[9] Weissman A, Jakobi P (1997). Sonographic measurements of the umbilical cord in pregnancies complicated by gestational diabetes. J Ultrasound Med.

[10] Barbieri C, Cecatti JG, Surita FG, Costa ML, Marussi EF, Costa JV (2011). Area of Wharton's jelly as an estimate of the thickness of the umbilical cord and its relationship with estimated fetal weight. Reprod Health.

[11] Begum K, Ahmed MU, Rahman MM (2016). Correlation between umbilical cord diameter and cross sectional area with gestational age and foetal anthropometric parameters. Mymensingh Med J.

[12] Raio L, Ghezzi F, Di Naro E, Franchi M, Maymon E, Mueller MD, Brühwiler H (1999). Prenatal diagnosis of a lean umbilical cord: a simple marker for the fetus at risk of being small for gestational age at birth. Ultrasound Obstet Gynecol.

[13] Richardson BS, Ruttinger S, Brown HK, Regnault TR, de Vrijer B (2017). Maternal body mass index impacts fetal-placental size at birth and umbilical cord oxygen values with implications for regulatory mechanisms. Early Hum Dev.

[14] Udoh BE, Erim A, Anthony E (2020). Sonographic assessment of umbilical cord diameter as an indicator of fetal growth and perinatal outcome. J Diagn Med Sonogr.

[15] Cromi A, Ghezzi F, Di Naro E, Siesto G, Bergamini V, Raio L (2007). Large cross-sectional area of the umbilical cord as a predictor of fetal macrosomia. Ultrasound Obstet Gynecol.

[16] Jarvis S, Glinianaia SV, Blair E (2006). Cerebral palsy and intrauterine growth. Clin Perinatol.

[17] Bilardo CM, Chaoui R, Hyett JA (2023). ISUOG practice guidelines (updated): performance of 11-14-week ultrasound scan. Ultrasound Obstet Gynecol.

